# The relation between obesity and breast cancer risk in women by considering menstruation status and geographical variations: a systematic review and meta-analysis

**DOI:** 10.1186/s12905-023-02543-5

**Published:** 2023-07-26

**Authors:** Tania Dehesh, Shohreh Fadaghi, Mehrnaz Seyedi, Elham Abolhadi, Mehran Ilaghi, Parisa Shams, Fatemeh Ajam, Mohammad Amin Mosleh-Shirazi, Paria Dehesh

**Affiliations:** 1 Modeling in Health Research Center, Institute for Futures Studies in Health, Kerman University of Medical Sciences, Kerman, Iran; 2 Department of Biostatistics and Epidemiology, School of Public Health, Kerman University of Medical Sciences, Kerman, Iran; 3 Department of Immunology, School of Medicine, Kerman University of Medical Sciences, Kerman, Iran; 4 Department of Health of Management and Medical Information Sciencese, Kerman University of Medical Sciences, Kerman, Iran; 5 Institute of Neuropharmacology, Kerman Neuroscience Research Center, Kerman University of Medical Sciences, Kerman, Iran; 6 Department of Anatomical Sciences, School of Medicine, Shiraz University of Medical Sciences, Shiraz, Iran; 7 Ionizing and Non-Ionizing Radiation Protection Research Center (INIRPRC), School of Paramedical Sciences, Shiraz University of Medical Sciences, Shiraz, Iran; 8 Department of Radio-Oncology, School of Medicine, Shiraz University of Medical Sciences, Shiraz, Iran; 9 Department of Epidemiology, School of Public Health, University of Medical Sciences, Tehran, Iran

**Keywords:** BMI, Breast, Cancer, Menopause, Breast neoplasms, Meta-analysis

## Abstract

Given the increase in the incidence of breast cancer during the past decades, several studies have investigated the effects of variables on breast cancer, especially obesity. This systematic review and meta-analysis aims to evaluate any effects of obesity on breast cancer risk in women, before and after menopause, and in different continents.

All forms of relevant literature examining any association between obesity and breast cancer, including cohort, case–control, and cross-sectional studies, were identified in the PubMed, Scopus, EMBASE, and Web of Science databases from January 1, 1990 until January 13, 2023. Body mass index (BMI) > 30 was used to indicate obesity. Every type of breast cancer was examined as outcome factors. The quality of the papers was evaluated using the Newcastle–Ottawa scale checklist. The Egger and Begg test was used to evaluate publication bias. To assess any extra impact of each research on the final measurement, a sensitivity analysis was carried out.

One hundred and two studies were included in this meta-analysis. Respectively, 48 and 67 studies reported associations between obesity and breast cancer in pre and post menopausal women. Combining all studies, the pooled OR of the association between obesity and breast cancer in pre-menopausal women was OR = 0.93 CI: (0.85–1.1), (I^2^ = 65.4%), and for post-menopausal woman, OR = 1.26 CI: (1.19–1.34), (I^2^ = 90.5%).

Obesity has a protective role in breast cancer among pre-menopausal women, but this relationship is statistically significant only in European women. The chance of developing breast cancer increases in post-menopausal women who are obese. This relationship is significant among Asian, North American, African and European women.

**Supplementary Information:**

The online version contains supplementary material available at 10.1186/s12905-023-02543-5.

## Introduction

The most frequent malignancy among women worldwide is breast cancer; 2.3 million new diagnoses of breast cancer in women, which represents 11.7% of all new cancer cases in the world, were made in 2020. Breast cancer, with 685,000 fatalities in a year, is the fifth cause of deaths among different cancers (1 in 6 cancer deaths) [1]. Environmental factors, including smoking, alcohol consumption, diet and eating habits, inactivity, obesity, excessive exposure to sunlight, and infection with some viruses and bacteria, have an important role in the emergence of breast cancer in females [2].

Obesity and overweightness are common and serious risk factors for many chronic diseases and cancers. According to the World Health Organization's (WHO) definition of obesity, people with a body mass index (BMI) > 30 are considered obese. The relationship between obesity and the occurrence of some chronic diseases and cancer has been clarified [3].

More than 50 research studies have looked into obesity and its role in the prognosis of breast cancer. A study revealed that the 5-year breast cancer survival rate for obese or overweight women was 55.6%, whereas it was 79.9% for women with normal weight. This study also demonstrated that obese women with breast cancer are more likely to have bigger tumors and invasive lymph nodes [4]. Many researchers have investigated the association between high BMI or obesity and developing breast cancer in women [5, 6], but there is some discordance between the findings of the studies. According to the findings of several observational studies, obesity raises the risk of getting breast cancer both before and after menopause [7–9]. The results of some studies show that obesity has a protective role in developing cancer in the pre-menopause period, while, it constitutes a risk factor in the post-menopause period [10, 11]. Pre-menopausal obesity in European and American women has been reported to decrease the risk of breast cancer [12, 13], while the positive association of pre-menopausal obesity with breast cancer incidence in Asian populations has been reported across the studies [14, 15].

It has been several years since systematic reviews and meta-analyses were done on the association between breast cancer and obesity. The meta-analysis study on the relationship between breast cancer and obesity needs to be updated as the numerous publications that have been published in recent years. According to our knowledge, no meta- analyse study has been conducted to investigate the relationship between breast cancer and obesity in different continents simultaneously. Since weight gain and breast cancer are both worldwide health concerns, this systematic review and meta-analysis aims to investigate the association between obesity and breast cancer risk in the pre- and post-menopause periods and also demonstrate any geographical variations by examining this association in different continents.

## Methods

The MOOSE and STROBE recommendations for reviews of observational studies were used in this systematic review [16, 17]. The protocol of this systematic review and meta-analysis has been recorded in Prospero (ID: CRD42021261448).

### Search method

Global databases including Scopus, Web of Sciences, Medline (PubMed), and EMBASE were searched to find all original published papers. No articles were found by searching through the Cochrane database. The papers listed in the retrieved studies were manually searched to increase search sensitivity. For January 1990 to January 2023, searches were carried out without linguistic constraints using "Obesity," "Body Weight," "Breast Neoplasms," "Breast Tumors," "Breast Carcinomas," and "Breast Cancer" as the the keywords. Only human observational studies were selected. Many of the articles from the original search results were eliminated after a review of the titles and abstracts. Two researchers separately identified the inclusion and exclusion criteria.

### Eligibility criteria

A study was not included unless it satisfied the following requirements: original publication, cross-sectional/case–control/cohort research, human studies, the main independent variable being obesity (BMI > 30), and the outcome being breast cancer. Review papers, editorials, letters, case reports, conference abstracts, studies that were not peer-reviewed, and articles on animals or genetics, were all eliminated from the study. All discrepancies found during the stages of data collection, compilation, and analysis were investigated and resolved by the authors.

### Data extraction

**Fig. 1:**
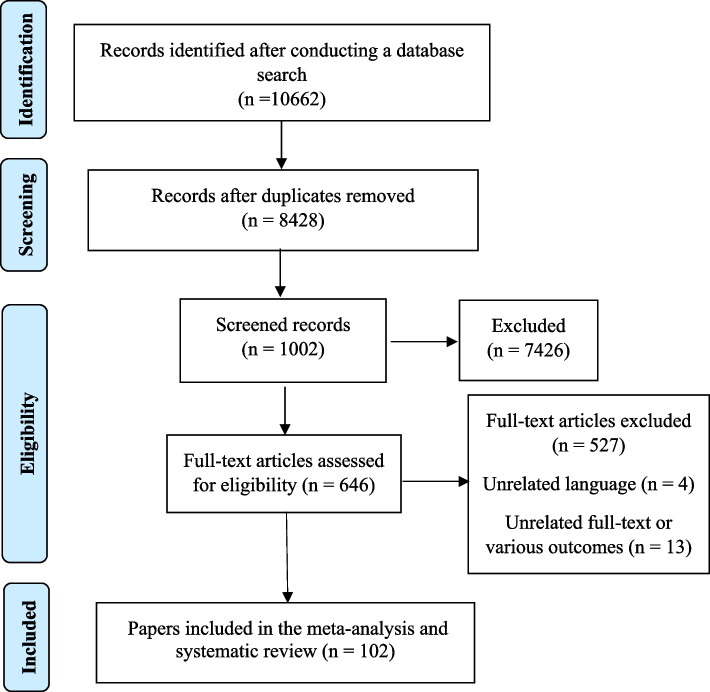
PRISMA Flow chart of the search and selection of the studies

Three researchers conducted separate evaluations of each included paper. A fourth author reviewed the work and helped them determine whether there were any differences of opinion. The two independent, matched reviewers who were responsible for gathering the data did so by using an Excel worksheet. Authors, publication year, study type, location, sample size, BMI, breast cancer type, and confonder variables of the association were listed in that order. After that, information from a systematic checklist was gathered. Figure 1 showed the PRISMA flowchart. In this flowchart, the number of initial search results in different databases was shown, and in the next step, the titles of the articles were checked, and finally, some articles were removed due to inappropriate titles and abstracts. According to the Fig. 1, about 10,662 articles were found in fiest setps search with sepecial keywords in international databases. Om the next setep, duplicates and articles that were not related to the topic in terms of title were removed, 1002 studies remained. In the next step, 527 articles were removed after reviewing the abstract, finally 102 articles were were entered into the meta-analysis.

### Risk of bias assessment

The obsevestional studies quality were evaluated by the Newcastle–Ottawa Scale (NOS) from 0 to 9 stars [18]. The articles based on the Newcastle–Ottawa Scale score were divided into three groups of 0–3 (fair), 4–6 (moderate), and 7–9 (good).

### Statistical analysis

This meta-analysis included all subtypes of breast cancer, regardless of clinical features or tumor stage. If several studies were published using the same data, the last study was selected and included in the meta-analysis [19]. The indicators used to measure the relationship in this meta-analysis were odds ratios (ORs) for case–control and cross-sectional studies, and risk ratios, relative risks, and hazard ratios for cohort studies. Most of the research conducted on the relationship between obesity and breast cancer were case–control studies, therfore, for better clarity, the OR has been considered as the primary index in this investigation. The results were determined according to the type of studies. ORs with standard error logarithm and logarithmic base were used for the meta-analysis. To estimate the pooled OR with a 95% confidence interval (CI 95%) due to heterogeneity between studies, random models were used using DerSimonian and Laird techniques [20]. Adjusted OR, where available, was preferred to sloppy measures. If several measures were reported in a study to investigate the association between obesity and breast cancer, the approach of combining the effect size reported in that study was used. If the heterogeneity test was significant, random effects models were employed to estimate the combined effect size. The Cochran's Q test and the I^2^ statistic were employed to determine statistical heterogeneity across papers in this research [21].

A meta-regression and subgroup analysis were also carried out to identify the reason for research heterogeneity. Moreover, the funnel plot and Egger and Begg's tests were performed to assess publication bias [22]. The trim and fill test was used to investigate publication bias further [23]. The statistical analysis was conducted by using Stata 14.0, and the threshold of statistical significance was established at *p* < 0.05.

## Results

### Identification and description of the studies

One hundred and two studies were included in this meta-analysis. Forty eight studies reported an association between obesity and breast cancer in pre-menopausal women, and 67 studies showed a relationship between obesity and breast cancer in post-menopausal women. 34 studies (4 cross-sectional,18 case–control, and 12 cohort) with a total sample size of 60,985 cases and 48,720 controls were used to evaluate the relation between obesity and breast cancer, without distinguishing between pre-menopausal and post-menopausal women.

Fifteen studies were conducted in North America, 21 in Asia, 8 in Europe, 2 in Africa, 1 in South America for the pre-menopause period. Twenty three studies were conducted in North America, 27 in Asia, 10 in Europe, 2 in Africa, 3 in South America and 1 in Oceania for the post-menopause period. We aimed to pool the findings of the research in this analysis individually depending on the subgroup of studies that measured the relation between obesity and breast cancer before and after menstruation. Table 1s describes the features of each study included in this systematic review and meta-analysis.

### Quality assessment and risk of bias

In this meta-analysis, the quality of case–control, cohort, and cross-sectional studies was examined by using the Newcastle–Ottawa scale (NOS), the results of which were presented in the checklists completed based on the type of study. The quality assessment checklists of the case–control and cohort studies showed that most of these studies had a moderate to high-quality score (Table 1s).

### Relationship between obesity and breast cancer risk in pre-menopausal women

**Fig. 2:**
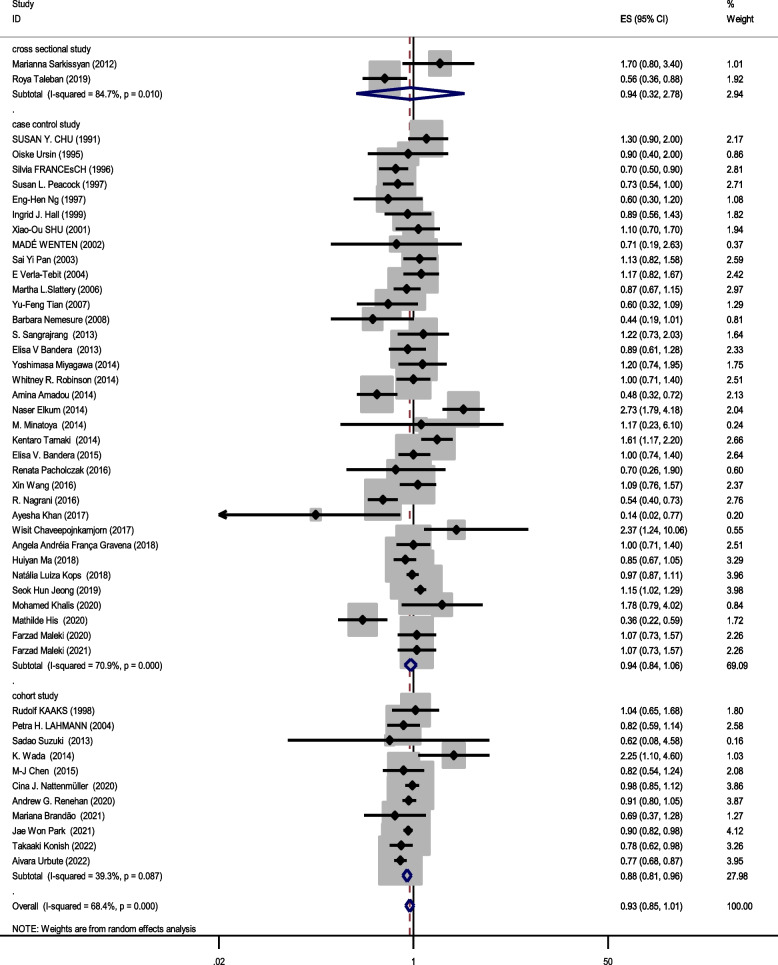
Meta-analysis of the relation between obesity and breast cancer risk in pre-menopausal women

Forty eight studies (two cross-sectional, 35 case–control, and 11 cohort) with a total sample size of 66,999 cases and 845,612 controls were included in the evaluation of the association between obesity and breast cancer in pre-menopausal women. Regardless of the type of study, the lowest and highest ORs were equal to 0.44 (95% CI; 0.19 – 1.01) and 2.73 (95% CI; 1.79 – 4.18), respectively. After combining all studies, the pooled OR of the association between obesity (with case group BMI > 30) and breast cancer in pre-menopause was OR = 0.93 CI: (0.85–1.1), I2 = 65.4%, *P* < 0.001 (Fig. 2). It showed that obesity was not a significant protective factor for breast cancer in pre-menopause women. Visual inspection of the funnel plot in Fig. 5a, indicated asymmetry, but Egger's tests (β =—0.071, *P*-value = 0.85), did not show significant publication bias. When the trim and fill approach was used to further evaluate publication bias, no study was imputed. The result of the meta-regression analysis described that the study quality and a sample size of more than 1000 had no significant linear relationship with the OR of obesity and breast cancer risk, so these variables were not the reason for the heterogeneity (Table 1).

**Table 1:** Findings of the meta-regression on the association between obesity and breast cancer risk

Study type	Variables	exp(b)	95% CI	P-value
**Pre-menopause**	Quality of study	0.91	(0.762–1.10)	0.37
	Sample size > 1000	0.85	(0.62- 1.16)	0.30
**Post-menopause**	Quality of study	1.03	(0.87- 1.20)	0.31
	Sample size > 1000	1.11	(0.68- 1.13)	0.71
**Regardless of menstrual status**	Quality of study	0.96	(0.79- 1.17)	0.71
	Sample size > 1000	0.84	(0.55- 1.29)	0.42

Sensitivity analysis showed that the pooled effect size on the relationship between obesity and breast cancer risk in pre-menopause women did not depend on a single study (CI range: 0.84- 1.01).

### Relationship between obesity and breast cancer risk in post-menopausal women

**Fig. 3:**
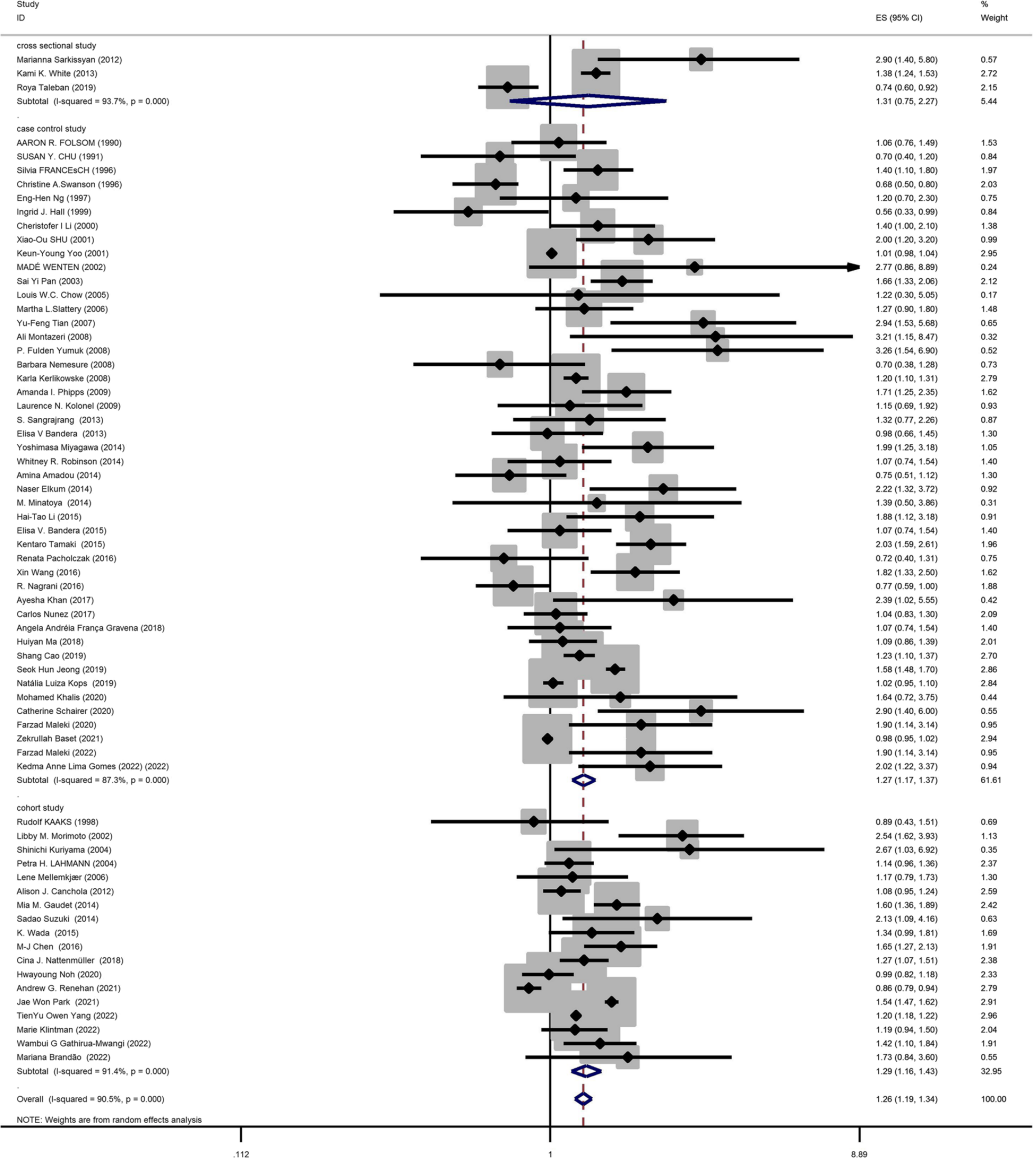
Meta-analysis of the relation between obesity and breast cancer risk in post-menopausal women

Sixty seven studies (three cross-sectional, 46 case–control, and 18 cohort) with a total sample size of 262,434 cases and 1,501,879 controls were used in the evaluation of the association between obesity and breast cancer risk in post-menopausal women. Regardless of the type of study, the lowest and highest ORs were equal to 0.56 (95% CI; 0.33 – 0.99) and 3.26 (95% CI; 1.54 – 6.9), respectively. After combining all studies, the pooled OR of the relation between obesity and breast cancer risk in post-menopausal women was OR = 1.26 CI: (1.19–1.34), I2 = 90.5%, *P* < 0.001(Fig. 3). It showed a higher probability of developing breast cancer by up to 26% in obese post-menopausal women. The funnel plots (Fig. 5b) for these studies provided substantial evidence of publication bias, but with the Egger test, a significant publication bias was not seen (β = 0. 78, *P*-value = 0.103). The trim-and-fill method used for more certainty (Fig. 6a), showed that 11 assumptive missing papers have been imputed, and the "adjusted" point estimate indicated a comparable OR compared to the original analysis (OR = 1.20, 95% CI: 1.13–1.27). The result of the meta-regression analysis indicated that the probability of having breast cancer in obese women increased with an increase in study quality and sample size, but this association was not statistically significant. Therefore, study quality and a sample size of more than 1000 were not the reasons for the heterogeneity.

Sensitivity analysis showed that the pooled effect size on the relationship between obesity and breast cancer risk in post-menopausal women did not depend on a single study (CI range: 1.18–1.34).

### Relationship between obesity and breast cancer risk regardless of menstrual status

**Fig. 4:**
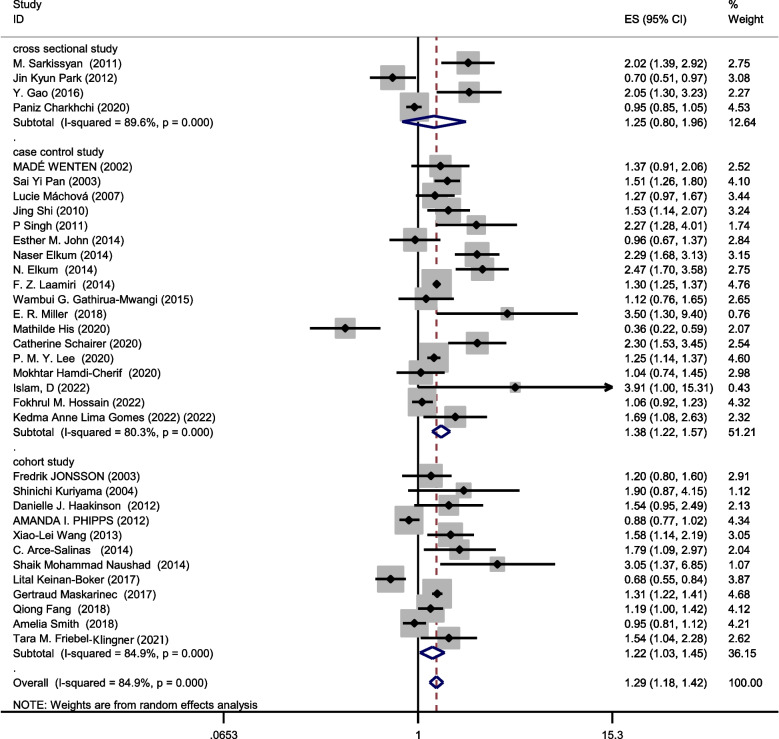
Meta-analysis of the relation between obesity and breast cancer risk in all women regardless of their menstrual status

**Fig. 5:**
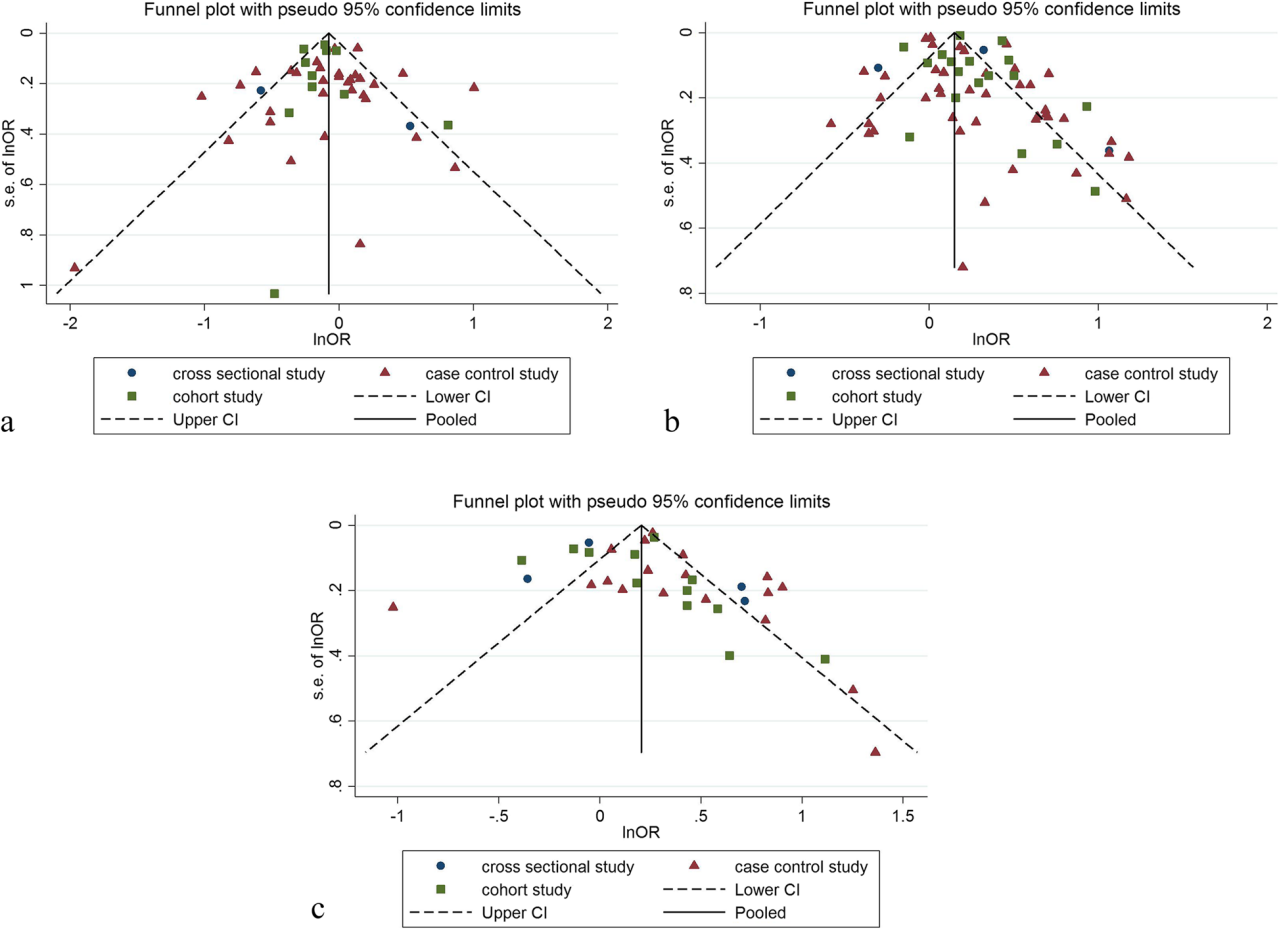
Funnel plots for publication bias in pre-menopause studies (**a**), in post-menopause studies (**b**) and studies regardless of menstrual status (**c**)

**Fig. 6:**
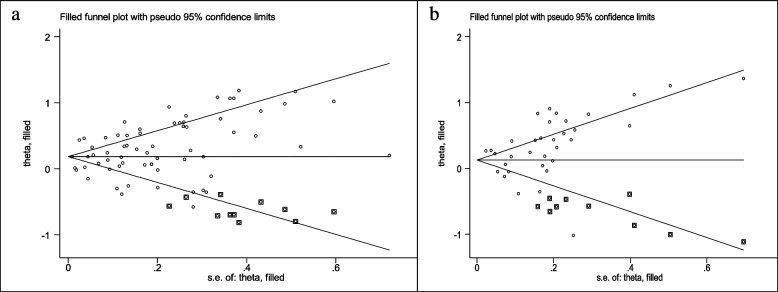
Trim-filled funnel plots for post-menopausal studies (**a**) and studies regardless of menstrual status (**b**)

The data from 34 studies (Four cross-sectional,18 case–control, and 12 cohort) with a total sample size of 34,962 cases and 2,076,826 controls were included in the evaluation of the relation between obesity and breast cancer risk, without distinguishing between pre-menopausal and post-menopausal women. After combining all studies, the pooled OR of the association between obesity and breast cancer was OR = 1.29 CI: (1.18–1.42), I2 = 84.9%, *P* < 0.001(Fig. 4). We observed an asymmetry based on a visual assessment of the funnel plot (Fig. 5c); however, no significant publication bias was indicated using Egger's test (β = 0.45, *P*-value = 0.75). Using the trim-and-fill procedure showed that 10 assumptive missing papers have been imputed, and after being included, the "adjusted" point estimate indicated a similar OR to the original analysis (OR = 1.13, 95% CI: 1.02—1.24) (Fig. 6b). According to the meta-regression analysis, study quality and a sample size of more than 1000 were not the reasons for the heterogeneity.

Sensitivity analysis indicated that the pooled effect size on the relationship between obesity and breast cancer risk did not depend on a single study (CI range: 1.17–1.42).

### Subgroup analysis based on combining menstrual status

Figures 2, 3 and 4 shows subgroup analysis irrespective of the type of study. The findings showed that obesity was linked to a decreased risk of breast cancer in pre-menopausal women across all study types. In the case–control papers, obese women had a 6% lower risk of breast cancer than normal-weight women (OR = 0.94, 95% CI: 0.84–1.06; I2: 70.9%; *P*-value 0.001), although this difference was not statistically significant. Also, in the cross-sectional studies (OR = 0.94, 95% CI: 0.32–2.78; I2: 84.7%; *P*-value < 0.001), obese women had a 6% lower risk of breast cancer than normal-weight women. There is a strong link between obesity and breast cancer in the pre-menopause period, according to cohort studies (OR = 0.88, 95% CI: 0.81–0.96; I2: 39.3%; *P*-value = 0.0.87). Case–control studies that explored the correlation between obesity and breast cancer risk in post-menopausal women reported that being overweight significantly increases the probability of developing the disease (OR = 1.27, 95% CI: 1.17–1.37; I2: 87.3%; *P*-value 0.001). It means that obese women have a 27% more chance of breast cancer in post-menopause relative to normal-weight women. Moreover, cross-sectional studies and cohort studies both showed a stronger link between obesity and breast cancer risk in post-menopausal women (OR = 1.31, 95% CI: 0.75–2.27; I2: 97.3%; *P*-value 0.001), and OR = 1.29, 95% CI: 1.16–1.43, respectively.

Combining the results of the studies that showed the relation between obesity and breast cancer risk regardless of menstrual status, the results of subgroups showed that case–control studies (OR = 1.38, 95% CI: 1.22–1.57; I^2^: 80.3%; *P*-value < 0.001), cohort studies (OR = 1.22, 95% CI: 1.03–1.45; I^2^: 84.9%; *P*-value = 0.0001) and cross-sectional studies (OR = 1.25, 95% CI: 0.8–1.96; I^2^: 89.6%; *P*-value < 0.001) report increased association between obesity and breast cancer risk.

Table 2 shows the results of the relation between obesity and breast cancer risk by geographic location of where the study was conducted. The result showed that Asian (OR = 1.49, 95% CI: 1.32–1.69; I2: 94.7%; *P*-value < 0.001) and North American (OR = 1.21, 95% CI: 1.07–1.36; I2: 75.5%; *P*-value < 0.001) obese woman in post-menopause were more likely to develop breast cancer than the European ones (OR = 1.10, 95% CI: 0.97–1.25; I2: 85%; P-value = 0.0001). The relation between obesity and breast cancer risk in post-menopause was not significant in European women. According to the different I^2^ tests in subgroup analysis based on geographic location, the difference in the geographical location of the studies is one of the reasons for the heterogeneity. Obesity was not a significant factor for breast cancer risk in pre-menopause in Asians (OR = 1.00, 95% CI: 0.87–1.15; I^2^: 76.71%; *P*-value < 000.1), and North Americans (OR = 1.07, 95% CI: 0.42–2.69; I^2^: 69.7%; *P*-value = 0.069). In European women, obesity was a significant protective factor for breast cancer risk in pre-menopause (OR = 0.88, 95% CI: 0.79–0.98 I^2^: 44.8%; *P*-value = 0.081).

**Table 2:** Association between obesity and the odds of breast cancer development by geographic location

	**Number of Studies**	**I**^**2**^	**P**^**2**^	**OR (95%CI)**	P-between*
**Association between obesity in pre-menopause and breast cancer risk**					
Asia	22	76.71	0.0001	1 (0.87–1.15)	0.0001
Europe	8	44.8	0.081	**0.88 (0.79–0.98)**	
Africa	2	69.7	0.069	1.07(0.42–2.69)	
North America	15	60.5	0.0001	0.84 (0.71–1)	
South America	2	0.0	0.896	0.97 (0.87–1.09)	
Oceania	-	-	-	-	
**Association between obesity in post-menopause and breast cancer risk**					
Asia	27	94.7	0.0001	**1.49 (1.32–1.69)**	0.0001
Europe	10	85	0.0001	1.10 (0.97–1.25)	
Africa	2	0.0	0.92	1.69 (0.98–2.92)	
North America	23	75.5	0.0001	**1.21 (1.07–1.36)**	
South Africa	3	70.8	0.033	1.21 (0.87–1.68)	
Oceania	1	0.0	-	1.04 (0.83–1.30)	
**Association between obesity and breast cancer risk regardless of menstrual status**					
Asia	14	86.1	0.0001	**1.45 (1.22–1.73)**	0.0001
Europe	3	0.0	0.87	**1.21 (1.01–1.46)**	
Africa	2	39.8	0.197	**1.24 (1.04–1.48)**	
North America	11	83.6	0.0001	**1.24 (1.05–1.46)**	
South America	3	92.5	0.0001	1.03 (0.37–2.54)	
Oceania	1	0.0		3.5 (1.3–9.41)	

^*^P-between: Heterogeneity between groups, P^2^: Heterogeneity between studies in group, I^2^:percentage of variation acrros studies due to heterogeneity

## Discussion

The breast cancer burden is substantial in terms of incidence, mortality and taking treatment resources. For example, radiotherapy centers normally deliver a large number of breast cancer treatments every day (usually larger than any other type of cancer) using a variety of techniques. [1, 24–26]. Any knowlwdge gained from detailed identification of risk factors that can be used toward improving breast cancer prevention is, therefore, highly useful and important. This systematic review and meta-analysis contributes to that effort.

The findings of this study divided the association between obesity and breast cancer risk into three categories: pre-menopause, post-menopause, and both together. This relation was also reported among the different continents in which the studies where conducted. The findings demonstrated that when combining those studies that did not separate the menopause period, obesity is shown to increase the risk of breast cancer. More specifically, obese women have a 1.29-fold higher chance of developing breast cancer than women of normal weight. The increased leptin in obese women has been associated with diagnosis of colon, breast, prostate and ovarian cancers in a previous study [27]. However, evidence for an association between obesity and breast cancer risk is controversial. Leptin is a fat-derived hormone that regulates appetite and energy homeostasis [28]. Studies have described that leptin receptors (LEPR) exist in breast cancer patients [29, 30].

Obesity not only increases the chance of breast cancer, but it also has a significant effect on the disease development process. Obese women with breast cancer are more susceptible to having larger tumors. These women may become resistant to hormone treatment and have a greater incidence of metastases. Clinical studies have shown that obese breast cancer patients receiving chemotherapeutic or estrogen inhibitors have a lower chance of recurrence than those who have normal weight [31, 32].

### Obesity and risk of breast cancer in pre-menopausal women

The dose–response meta-analysis conducted by Liu et al. reported that a 5 unit increase BMI leads to a 2% increase in the probability of breast cancer. Also, that study's findings revealed that women with greater BMIs before menopause had a lower chance of getting breast cancer [6]. The results of the current meta-analysis also show that obesity is a protective factor against breast cancer in pre-menopause.

It has been demonstrated that increased endogenous estrogen exposure in women raises the probability of developing breast cancer. Therefore, long-term exposure to estrogen for various reasons such as early-age menstruation or prolonged menopause may raise the probability of developing breast cancer [33]. Due to a possible hormonal interaction during ovulatory cycles, young obese women have a higher reduction in progesterone levels at the end of the menstruation cycle compared to the normal woman, and progesterone levels come down later in obese women [34]. Obese pre-menopausal women have longer periods than women who have normal weight before menopause. As a result, the duration of exposure to estrogen in these people is less, which could reduce the chance of breast cancer in these people [33].

### Obesity and risk of breast cancer in post-menopausal women

According to the results of several studies, post-menopausal obesity increases the risk of breast cancer. This may be due to producing more estrogen hormone from adipose tissue in obese women in post-menopause [35].

The results of several studies show that the serum level of sex hormones such as estrogen is higher in women with breast cancer compared to healthy women of the same age [36–38]. Reduction in sex hormone-binding globulin receptors leads to a higher level of estrogen in obese women compared to normal-weight women in post-menopause [33]. As a result, it is thought that the primary location of estrogen manufacture in obese post-menopausal women occurs in excess adipose tissue, resulting in a multiplication of the estrogen hormone in the blood. Thus, increasing estrogen levels may increase the probability of breast cancer in post-menopausal women [33].

### Subgroup meta-analysis

After performing the subgroup analysis in each continent, it was found that obesity during pre-menopause is a significant protective factor among the population of European women. This protective relation was also found in the other continents, but without statistical significant. The findings of this study are in contrast with those of another meta-analysis that identified a significant positive relation between obesity and breast cancer risk in pre-menopausal Asian women [39]. The dose–response meta-analysis revealed that every 5 kg/m^2^ increase was significantly and negatively associated with the risk of breast cancer during the pre-menopause period among Africans (RR = 0.95, 95% CI: 0.91, 0.98) [15].

Throughout this meta-analysis, heterogeneity was found in studies that show the relation between obesity and breast cancer risk in pre- and post-menopausal women. This heterogeneity was found in different study designs. The relation between breast cancer risk and obesity may have been confounded by a factor such as the status of taking hormonal drugs, the type of breast cancer, geographical location, sample size, physical activity, etc. To investigate the sources of heterogeneity observed among the studies, subgroup analyses, and meta-regression were performed for some of these confounding factors. The results of subgroup analyses showed that geographical location may be one of the reasons for heterogeneity. The meta-regression results showed that sample size and the quality of the studies do not have a significant effect on creating heterogeneity.

### Strengths

One of the strengths of this meta-analysis compared to previous meta-analyses is combining different study designs separately and performing sensitivity analysis. Also, the results of the relationship between obesity and breast cancer risk have been reported separately before and after menopause.

### Limitations

There were several limitations in our study. First, obesity was calculated using BMI by measuring height and weight at the time of diagnosis. The association between obesity and breast cancer did not take into account long-term variations in weight and body composition, and several other weight-determining factors (including waist circumference and waist-to-hip ratio). Second, BMI grouping in some articles was not based on WHO standards. Third, the type of breast cancer was not specified in many studies. Fourth, few studies have been done on obesity and breast cancer risk in Oceania, South America, and Africa. Therefore, generalizing the results of this meta-analysis in these continents should be done with caution. Finally, important confounders of breast cancer and obesity were ignored in some studies and this may affect the result of the current analysis.

## Conclusions

Obesity has a protective role in breast cancer among pre-menopausal women, but this relationship is statistically significant only in European women. The chance of developing breast cancer increases in post-menopausal women who are obese. This relationship is significant among Asian, North American, African and European women. Therefore, it is recommended that post-menopausal women prevent overweight and obesity by having sufficient physical activity and suitable nutrition.

## Supplementary Information

**Additional file 1.** 

## Data Availability

The datasets used and/or analysed during the current study are available from the corresponding author on reasonable request.
