# Supplementary material for: The relation between obesity and breast cancer risk in women by considering menstruation status and geographical variations: a systematic review and meta-analysis
Source: BMC Womens Health. 2023 Jul 26;23:392. doi: 10.1186/s12905-023-02543-5 (PMC10373406; doi:10.1186/s12905-023-02543-5)
Supplement: Supplementary file 1 — Additional file 1. [file 12905_2023_2543_MOESM1_ESM.docx]

|  | Table 1S: The main characteristics of included studies of the association of obesity and breast cancer | | | | | | | | | |
| --- | --- | --- | --- | --- | --- | --- | --- | --- | --- | --- |
| **Authors (Years)** | | **Country** | **Type of Studies** | **Study Population (Age/sex)** | **Sample size (Case/ Control and Exposure/Non-exposure)** | **Effect Size (Adjusted OR [%95 CI] or Adjusted RR [%95 CI])** | **Adjusted variables** | **Type of cancer** | **NOS score** |  |
| Cina J. Nattenmüller (1)  (2018) | | Germany | prospective cohort | 30,270 (35-65 years/women) | postmenopausal  136case/  5815control  pre- and perimenopausal:  308case/  15683control | Among postmenopausal HT:  HR: 1.27 (CI: 1.07, 1.50)    pre- and perimenopausal:  HR: 0.98 (0.85, 1.12) | 12,9,11, 16, | aggressive tumor subtypes.  (ER, PR, HER2, Ki67, Bcl-2 and p53) | 8 |  |
| C. Arce-Salinas (2) (2014) | | Mexico | retrospective cohort | 819  (49 years ± 11.18 /women) | (596 overweight and obesity /  223 normal weight) | HR: 1.79 (1.09–2.97) | 1, 17, 18, 19, 20, 21,22 | locally advanced breast cancer  (LABC) | 9 |  |
| Elisa V Bandera (3)  (2013) | | USA | case–control | 1936  (age 20–75/ women) | Pre-menopausal:  (469 cases / 482 controls)  Post-menopausal:  (509 cases / 476 controls) | Pre-menopausal:  OR: 0.89 (0.61–1.28)  Post-menopausal:  OR: 0.98 (0.66–1.45) | 1, 23, 13, 11, 2, 3, 4, 5, 8, 10, 7 | early/aggressive breast cancer  carcinoma in situ or invasive breast cancer | 8 |  |
| M-J Chen (4) (2015) | | Taiwan | Prospective  cohort | 1,393,985 women  (503,007 women were pre-menopausal, and 890,978 women were post-menopausal) | pre- menopause = (8571 cases/494436)  post- menopause=  (10716 cases/880262) | pre menopause  HR= 0.82( 0.54-1.24)  post menopause  HR=1.65( 1.27 -2.13) | 1, 2, 4, 9, 10, 8 | ate-onset breast cancer | 8 |  |
| Qiong Fang (5) (2018) | | China | Retrospective Cohort | 3080 (24–87 years /Female) | (1540 cases/1540 controls)  (679 obesity/2401 no obesity) | OR: 1.19 (1.00–1.42) | 1 | - | 6 |  |
| Danielle J. Haakinson (6) (2012) | | USA | Retrospective Cohort | 1352  (20-95 /women) | (327 obese/ 1025 nonobese) | HR: 1.54(0.95 -2.49) | 1 | invasive breast cancer | 6 |  |
| Lital Keinan-Boker + 4(7)  (2016) | | Israel | Cohort | 951,480 (16–19 years/women) | 947,689  (9,619 case/ 938070 control) | HR = 0.682 (0.552–0.843) | 13, 11, 12 | - | 8 |  |
| Natália Luiza Kops (8) (2018) | | Brazil | nested case-control | 216  (60.3 ± 10.4 vs. 55.8 ± 8.4 years/ women) | Premenopausal:  (19case/53control)  Post-menopausal:  (38case/106control) | Premenopausal: OR=0.97 (0.84 - 1.11)  Post-menopausal:  OR= 1.02 (0.95 - 1.10) | 1, 14, 15 | - | 8 |  |
| Gertraud Maskarinec (9)  (2017) | | USA | Cohort | 103,721 (aged 45-75 years /Women) | 896 (424 obesity, 472 no obesity) | HR:1.31 (1.22-1.41) | 4,1 ,11, 2, 25, 26, 24, 6, 21, 23 | breast cancer, including in-situ and invasive tumors | 7 |  |
| R. Nagrani (10)  (2016) | | India | case-control | premenopausal: 1659  postmenopausal women: 1478  age; 20-69 | premenopausal:  (818 cases and 841 controls)  Postmenopausal:  (815 cases and 663 controls) | premenopausal: OR = 0.5 (0.4-0.8)  postmenopausal:(OR =1.8 (1.1-3.3)) | 1,13 | breast cancer irrespective of hormone receptor (HR) status | 7 |  |
| Shaik Mohammad Naushad (11) (2014) | | India | case–control | 593 women | (case=341/control=252) | OR = 3.05  (1.37–6.85) | - | Breast cancer | 7 |  |
| AMANDA I. PHIPPS (12) (2012) | | USA | cohort | 1,054,466 (ages 40 to 84 years/ women) | (13797case/ 1040669 controls) | HR= 0.88(0.77-1.02) | 1 | 1)ER-positive (ERþ, n Z 10,026)  2) HER2-expressing (ER-negative/PR-negative/HER2-positive, n Z 308) 3)triple-negative (ER-negative/ PR-negative/HER2-negative, n Z 705). | 7 |  |
| Amelia Smith (13)  (2018) | | USA | Cohort | 3304 women with stage I-III breast cancer (63-73age) | (758 obesity, 2253 non obesity) | Multivariable:  RR: 0.95 (0.81, 1.12) | 1, 27, 28, 22, 18, 20 | lymph node positive breast cancer | 8 |  |
| Sadao Suzuki (14)  (2013) | | Japan | prospective cohort | 36 164 (aged 40–79 years/ women) | 234cases/  35930controls | Premenopausal:  HR:0.62  CI: (0.08–4.58)  Postmenopausal:  HR: 2.13, (1.09–4.16 ) | 1, 12, 4, 5,11, 24, 14, 21, 2, 16, 25, 26, 13. | - | 8 |  |
| Kentaro Tamaki (15)  (2014) | | Japan | Case-control | (9006)  median ages were 53 years old (20-99) of case/ 44 years old (20-90) of control | (3431/5575)  Premenopausal:  (59case/140control)  Postmenopausal:(  (181case /109control) | Premenopausal:  OR=1.61 (1.17-2.20)  Postmenopausal:  OR=2.03 (1.59-2.61) | - | - | 7 |  |
| K. Wada (2014) (16) | | Japan | Cohort | 234325 women | Premenopausal cancer: (1148 expose/ 55265 non expose)  Postmenopausal cancer: (4713 expose/173199 non expose) | Premenopausal: RR=2.25 (1.10–4.60)  Postmenopausal:  RR=1.34 (0.99–1.81) | 1, 13, 16, 25, 4 | - | 8 |  |
| Xiao-Lei Wang (17)(2013) | | China | case-control | 492 (49.94 mean age/ women ) | (123 cases/ 369 control) | OR = 1.58,  (1.14 -2.19) | 2,3,29,30 | - | 7 |  |
| Kami K. White(18) (2013) | | Hawaii and Los Angeles | Case-control | 82971 men , women  45–75 years. | (3080 case/79891control) | Postmenopausal: hazard ratio = 1.38, (1.24-1.53), | 1,2,31,4,5,9,16,25,32 | invasive breast cancer |  |  |
| Zekrullah Baset(19)(2021) | | Afghanistan | Case-control | 402 | (201 case/  201 control) | Postmenopausal: Odds Ratio = 0.98, (0.95-1.02) | - | - |  |  |
| Ayesha Khan(20)  (2017) | | Pakistan | case control | CASE= breast cancer: 56 women  Mean age=  46.93(SD=10.2  postmenopausal  =45  pre-menopausa  =11  Control=168 women free of breast diseas  mean age= 44.60years (SD=12.2 | CASE=39  CONTROL=109 | pre-menopausal women (OR=0.14; 95% CI: 0.02-0.77)  post-menopausal women (OR=2.39; 95% CI: 1.02–5.55) | - | - | 9 |  |
| Hai-Tao Li(21)  (2015) | | China | Case- control | Case=  206 female patients aged 27-86(median age of 53 years old)  Control=  210 healthy individuals  aged 28-85(median age of 53-year old) | (206=case/210 control) | Postmenopausal  OR=1.88, (1.12-  3.17) | 1 | - | 8 |  |
| Yoshimasa Miyagawa(22)  (2013) | | Japan | case–control | 1,297 Japanese women  Mean age=57.9 years(standard deviation (SD) 13.2, range 20–92) | Premenopausal women  (223 case/97Control)  Postmenopausal women  (392 case/131Control) | pre-menopausal women : OR= 1.2, (0.74 1.95)  post-menopausal women :OR=1.99, (1.25 3.18) | 1,4,5,2,24 | Luminal A  Luminal B | 8 |  |
| Shang Cao(23)  (2019) | | China | Case–control | 1651 women | Premenopausal women: (254 case/362 control)  Postmenopausal women: (518Case/  517 Control) | pre-menopausal women : OR= 2.39,  ( 1.02-5.55)  post-menopausal women :OR=0.14, ( 0.02,0.77) | 1,4,11,6,24,5,2,3,7,31,26,25,12,33 | - | 9 |  |
| Jing Shi(24)  (2010) | | china | case-control | 1233 under 75 years of age(<40- ≥70)  Women | (643 cases/590 controls) | OR=1.53,( 1.14, 2.07) | 1,11,13,16,25,24,7,34,3 |  |  |  |
| Yu-Feng Tian(25)  (2007) | | Taiwan | case–control | Case=244  age range of 22–87 years  mean age= 51.5 (11.4)  Control=244  Mean age= 48.4 (7.9) | premenopausal :  (141 cases/141 controls)  postmenopausal:  (103 case/103 controls) | pre-menopausal women : OR= 0.6,  ( 0.32-1.09)  post-menopausal women :OR=2.94,( 1.53-5.68) | 1,8,34, | Overall, 44 of the cases were  in situ and 195 of the cases were invasive.  150 (73%) were ER-positive and 55 (27%) were ER-negative. |  |  |
| E Verla-Tebit(26)  (2004) | | Germany | case–control | 1674 women | premenopausal women: (558 cases/1116 controls) | pre-menopausal women : OR= 1.17, ( 0.82-1.67) | 4,7,24,2,8,25 |  |  |  |
| Esther M. John(27)  (2014) | | America | case-control | 2,023 Hispanic and 2,384 NHW women  :aged 35–79 years | (1,715 cases/2,108 controls) | OR=0.96, ( 0.67, 1.37) | 1,23,11,2,4,9,8,6,25 |  |  |  |
| Temidayo O.Ogundiran(28)  (2012) | | Nigeria | case-control | 384 women | (194case/194contr)ol | OR=1.63,( 1.06-2.5) | 23,11,4,9,6,8,33,2,3,31,12,25 |  |  |  |
| Louis W.C. Chow(29)  (2005) | | China | case control | 551 women aged 24–85 years | (198 cases/353 control) | Postmenopausal: OR=1.22, (0.3-5.05) | 1 |  |  |  |
| Libby M. Morimoto(30)  (2002) | | United States | cohort | 85,917 postmenopausal women aged  50–79. | (1030 cases/84887 controls) | Postmenopausal: RR=2.54,( 1.62-3.93) | 1,11,5,24,6,2,16,4,23,25,26,32 |  |  |  |
| Xiao-Ou SHU(31)  (2001) | | China | case-control | 3015 women (25 to 64) years of age | Premenopausal women:  (952 case/ 990 control)  Postmenopausal women:  (501cases/ 562 control) | Pre menopausal: OR=1.1,( 0.7-1.7)  Postmenopausal: OR=2, ( 1.2-3.2) | 1,2,11,4,6,5 |  |  |  |
| Keun-Young Yoo(32)  (2001) | | Japan | Case- control | All cases and controls were 25  years of age or older | (1154 cases /21 714 controls). | Postmenopausal: OR= 1.07, (1.04, 1.1) | 1,2,4,5,6,9,8,12,16,25 | estrogen (ER) and/or progesterone receptor (PR) status in the breast tissue was evaluated |  |  |
| Cheristofer I Li(33)  (2000) | | USA | Case- control | Middle age postmenopausal women  50-64 years old | 4(79 cases/435 controls) | Postmenopausal: OR= 1.4,( 1-2.1) | 1,2,24 |  |  |  |
| Ingrid J. Hall(34)  (1999) | | Amrica | case-control | Black women and White women, aged 20-74 years | Black women: (350 cases/ 353 controls) and White women: (523 cases/ 471 controls) | Pre menopausal: OR= 0.89,( 0.56-1.43)  Postmenopausal: OR= 0.56(0.33-0.99) | 1,4,5,11,24,35 |  |  |  |
| Susan L. Peacock(35)  (1997) | | Washington State | case-control | women aged 21-45 years  845 cases  961control | )845 cases/  961controls) | Pre menopausal: OR= 0.73, ( 0.54-1) | 1,4 |  |  |  |
| Rudolf KAAKS(36)  (1998) | | Netherlands | COHORT | 11,663 participants | Pre menopausal: (29 cases/5869controls)  Postmenopausal:(30cases/3491controls) | Pre menopausal: OR= 1.04, (0.65-1.68)  Postmenopausal: OR= 0.89, (0.43-1.51) |  |  |  |  |
| Eng-Hen Ng(37)  (1997) | | Singapore | case-control | (204 cases and 882 controls)  women  45to69 years old | Premenopausal:  (74cases/297 controls)  Postmenopausal:  (130 cases/585 controls) | Pre menopausal: OR= 0.6,( 0.3 -1.2)  Postmenopausal: OR= 1.2,( 0.7-2.3) |  |  |  |  |
| Silvia FRANCEsCH(38)  (1996) | | Italy | case-control | 2,569 cases aged 23-74 years (median age,55 years)  Control: 2,588 women aged 20-74 years (median age, 56 years) | Premenopausal:  (105cases/113 controls)  Postmenopausal:  (392 cases/401 controls) | Pre menopausal: OR= 0.7,( 0.5 -0.9)  Postmenopausal: OR= 1.4,( 1.1-1.8) |  |  |  |  |
| Christine A.Swanson(39)  1996 | | United States? | case-control | women aged 20-44 years | (320cases/310controls) | Pre menopausal: OR= 0.68,( 0.5-0.8) | 1,23,4,7,24, |  |  |  |
| Oiske Ursin(40)  1995 | | USA | Case- control | 142 premenopausal  bilateral breast cancer cases  229 sister controls | (31 cases/63 control) | Pre menopausal: OR= 0.9,( 0.4-2) | 1,11,25,7,4 |  |  |  |
| AARON R. FOLSOM(41)  1990 | | USA | Cohort | postmenopausal women aged 55-69 years | (229cases/ 1,839controls) | Postmenopausal: OR= 1.06,( 0.76-1.49) | 1 |  |  |  |
| Whitney R. Robinson(2014) | | USA | Case- control | Case women aged 20–74 years  control women aged 65–74 years | Black :(788 cases/718 controls)  White: (995 cases/818 controls) | Pre menopausal: OR= 1, (0.71-1.4)  Postmenopausal: OR= 1.07,( 0.74-1.54) | 1,2,25,4,24,6 |  |  |  |
| Mohamed Khalis(42)  (2020) | | Morocco | Case-control | 300 cases of breast cancer and 300 matched controls | Premenopausal women:  (54 cases/46 controls)  Postmenopausal women: (64 cases/ 51 controls) | Pre menopausal: OR= 1.78 (0.79–4.02)  Postmenopausal: OR= 1.64 (0.72–3.75) | 1,13,9,7,8,6,26 |  |  |  |
| Roya Taleban(43)  (2019) | | Iran | cross-sectional study | 7,805 women | Pre menopausal: (42 cases/1049 controls)  Postmenopausal:  (275 cases/878 controls) | Pre menopausal: OR= 0.56, ( 0.36-0.88)  Postmenopausal: OR= 0.74, (0.6-0.92) | 1,4,6,9,11,2,7,31,25, 16 |  |  |  |
| Angela Andréia França Gravena(44)  (2018) | | Brazil | Case-control | 500, postmenopausal women | Pre menopausal: (12 cases/27 controls)  Postmenopausal:  (23 cases/60 controls) | Pre menopausal: OR=1, ( 0.71-1.4)  Postmenopausal: OR= 1.07,( 0.74-1.54) | 1,4,7 | estrogen receptor (ER) subtype |  |  |
| Wisit  Chaveepojnkamjorn(45),  2017 | | Thailand | Case-Control | Premenopausal Women  257 cases and 257 controls  average age=39 years | (26 cases/ 10 control) | Pre menopausal: OR=2.37, (1.24-10.06) | 2,3,4,7,16 |  |  |  |
| Renata Pacholczak(46)  2016 | | Poland | Case-control | 487women—193 cases  aged between 35 and 80 years  and 294 control | ALL: (56 cases/63 controls)  Premenopausal women: (12 cases/25 controls)  Postmenopausal women: (44 cases/38 controls) | ALL=0.72 (0.40–1.31)  Premenopausal women: 0.70 (0.26–1.90)  Postmenopausal women: 0.49 (0.20–1.19) |  |  | 8 |  |
| Elisa V. Bandera(47)  2015 | | Africa  AMERICA  N | case-control | 2,104 ER+ cases, 1,070 ER- cases (including 491 TN cases), and 12,060 controls | Premenopausal (221case/835 controls)  Postmenopausal: (455 cases/1460 controls) | Premenopausal women: 1,( 0.74-1.4)  Postmenopausal women: 1.07,( 0.74-1.54) | 1,11,13,2,4,24,8,6,31,7,5 |  | 9 |  |
| Huiyan Ma(48)  2018 | | USA | case-control | 6320 women (3934 case-patient participants, 2386 control  aged 35–64 years | Premenopausal  (200cases/349 controls)  Postmenopausal: (263cases/325controls) | Premenopausal women: 0.85,( 0.67-1.05)  Postmenopausal women: 1.09,( 0.86-1.39) | 13,23,11,1,2,4,9,8,26,25,16,7 |  | 7 |  |
| Mathilde His(49)  2020 | | Colombia | multicentric population-based case-control study | Case=406  Mean age=38.7 (5.1)  Control=406  Age=38.6 (5.2) | (72Case/133control) | OR=0.36,( 0.22-0.59) | 11,2,3,26,6,9,8,4,16,24,12,36 |  | 8 |  |
| Paniz Charkhchi(50)  2020 | | United States | cross-sectional survey | **breast cancer screening, n = 163,164;**  women  ,age =  40-75 years | (54987cases/108177) | OR=0.95,( 0.85-1.05) | - |  | 6 |  |
| Seok Hun Jeong(51)  2019 | | Korea | Case–control | 16,190 female breast cancer:  aged between 35  and 80 years  Control=32380 | Premenopausal  (1611cases/211controls)  Postmenopausal: (1823cases/364controls) | Premenopausal women: 1.15,( 1.02-1.29)  Postmenopausal women: 1.58,( 1.48-1.7) | 1,2,4,8,6,7,9,37 | Luminal A =6272  HER2− Luminal B =  2462  HER2+ Luminal B=  1839  HER2− express=  2361  Triple negative=  3256 | 9 |  |
| P Singh(52)  2011 | | India | matched case-control | 320 breast cancer patients .  mean age=45.5 years  320 normal  mean age=40.98 years  women | (43 cases/21 control) | OR=2.27 (1.28–4.01) | 1,18,20 |  | 7 |  |
| Ali Montazeri(53)  2008 | | Iran | case-control | postmenopausal women  The mean age for cases was 54.6 (SD = 6.8) and 52.7 (SD = 6.8) for controls | Postmenopausal (116cases/116 controls) | Postmenopausal OR= 3.21,( 1.15-8.47) | 1,2,4,24 |  | 8 |  |
| Amanda I. Phipps(54)  2009 | | Washington | case-control | Controls :n=1,447  Cases: n=1124    Age=55-79 | Postmenopausal (221 cases/197 controls) | Postmenopausal OR= 1.71,( 1.25-2.35) | 1 | Luminal (ER+)  HER2−Overexpressing (ER−/PR−/HER2+) Triple-Negative (ER−/PR−/HER2−) | 8 |  |
| P. Fulden Yumuk(55)  2008 | | Turkey | case-control | All Case=( 1172 females: 423 pre- and 749 postmenopausal,  and 843 males, aged> 19 years)  and median age was 57 years (range 20-84) | Postmenopausal (321 cases/29controls) | Postmenopausal OR= 3.26,( 1.54-6.9) |  |  | 7 |  |
| Martha L.Slattery(56)  2006 | | USA | Case-control | Cases (n = 2,325)  controls (n = 2,525)  Age=25---79 | Premenopausal  (188cases/197controls)  Postmenopausal: (167cases/216controls) | Premenopausal women: 0.87,( 0.67-1.15)  Postmenopausal women: 1.27( 0.9-1.8) | 1,12,26,24,25,4,6,13 | Non-Hispanic White:  Hispanic/AIAN: |  |  |
| Lene Mellemkjær(57)  2006 | | Denmark | Cohort | 23,788 Postmenopausal Women  19≥ ---- +35 | (35 cases/13193controls) | Postmenopausal women: 1.17,( 0.79-1.73) | 24,6,3,11,25 | Never HRT users (N=11,796)  Ever HRT users (N = 11,992 | 8 |  |
| Jin Kyun Park(58)  2012 | | Kore | cross-sectional | 5213 women aged between 40 and 80 years old |  | Postmenopausal women: 0.7,( 0.51-0.97) |  |  | 6 |  |
| Marianna Sarkissyan(59),  2012 | | USA | cross-sectional | Cases=237 (50.3)  control=234 (49.7)( greater than 30 years) | Premenopausal  (144cases/119controls)  Postmenopausal: (144cases/119controls) | Premenopausal women: 1.7,( 0.8-3.4)  Postmenopausal women: 2.9( 1.4-5.8) |  |  | 6 |  |
| Barbara Nemesure(60)  2008 | | USA | case–control | black population:  cases :  mean (SD) ages of 57 (14.3) years  controls(at least 21 years of age):  Mean (SD) age of 56 (14.1) years | Premenopausal  (24cases/67controls)  Postmenopausal: (49cases/110controls) | Premenopausal women: 0.44,( 0.19-1.01)  Postmenopausal women: 0.7,( 0.38-1.28) | 1,2,3,4,6,26,24,31 |  | 8 |  |
| Lucie Máchová(61)  2007 | | Czechia | case-control | 5,700 cases and 37,772 controls,  aged 30–64 years | (554 cases/20776 controls) | OR=1.27, (0.97-1.67) | 1,14,16,38 |  | 8 |  |
| Amina Amadou(62)  2014 | | Mexico | case–control | One thousand incident cases and 1074 matched control women aged 35–69 years | Premenopausal:  (133cases/202 controls)  Postmenopausal:  ( 257 cases/314 controls) | Premenopausal women: 0.48 (0.32, 0.72)  Postmenopausal women: 0.75 (0.51, 1.12) | 1,13,8,6,24,25,26,2,14,32 |  |  |  |
| Mia M. Gaudet(63)  2014 | | USA | Cohort | 28,965 postmenopausal women. | (1088cases/ 28965 controls) | Postmenopausal women: 1.6,( 1.36-1.89) | 14,11,24,6,16,23,24,2,7,36,5,26,3,31 |  | 9 |  |
| Alison J. Canchola(64)  2012 | | America | Cohort | Among 52,642 postmenopausal women,  Age at baseline (yrs)= 62 (56 – 70) | (218 cases/740 control) | Postmenopausal women : OR=1.08,( 0.95-1.24) | 2,3,4,6,,25,31, | 2,321 developed invasive breast cancer with known estrogen- and progesterone-receptor status (1,652 ER+PR+, 338 ER+PR−, 312 ER−PR−) | 9 |  |
| Shinichi Kuriyama(65)  2004 | | Japan | cohort | 27,539 persons (668 women with breast cancer) aged 40 years or older. | All; (7 cases/73 controls)  Postmenopausal women: (5case/37 controls) | OR=1.9,( 0.87-4.15)  Postmenopausal women : OR= 2.67,( 1.03-6.92) | 1,16,25,24,4,6 |  | 8 |  |
| Petra H. LAHMANN(66)  2004 | | 10 European countries: Denmark, France, Germany,  Greece, Italy, the Netherlands, Norway, Spain, Sweden and the  United Kingdom | cohort | The analytic cohort  therefore consisted of 176,886 women from 9 countries, 73,542  premenopausal and 103,344 naturally postmenopausal. | Premenopausal:  (68cases/132controls)  Postmenopausal:  ( 181 cases/350controls) | Premenopausal women: 0.82,( 0.59-1.14)  Postmenopausal women: 1.14,( 0.96-1.36) | 1,13,11,16,25,24,6,4 |  | 8 |  |
| Sai Yi Pan(67)  2003 | | Canada | case-control | 21,022 incident cases of 19 types of cancer  and 5,039 controls aged 20–76 years.  Women with breast cancer =9522 controls=2492 | Premenopausal:  Case=913  Postmenopausal:  Case=1,449  All: 2364 cases | Premenopausal women: 1.13 (0.82, 1.58)  Postmenopausal women: 1.66,( 1.33- 2.06)  All: 1.51 ,( 1.26- 1.80) | 4,6,9,34 |  | 8 |  |
| Fredrik JONSSON(68)  2003 | | Sweden | COHORT AND CO-TWIN  CONTROL STUDIES | 21,884 Swedish twins born  breast cancer(n = 607).  The median age at baseline was 56 years (range 44 – 83). | (40 cases/19374) | OR=1.2,( 0.8-1.6) | 1 |  | 8 |  |
| MADÉ WENTEN(69)  2002 | | America | case-control | New Mexico of Hispanic (n  694) and non-Hispanic (n 813) white women.  (30–74 years) | All:(71cases/68 controls) | OR=1.89 (1.02–3.50)  Premenopausal women: 0.71 (0.19–2.63)  Postmenopausal women: 1.66,( 1.33- 2.06) | 1,2,24,7,8,9,31 |  | 9 |  |
| SUSAN Y. CHU(70)  1991 | | USA | case-control | Case=4323 women aged 20-54 years  Control=4358 women | Premenopausal women: (2053 case/1759 controls)  Postmenopausal women: (773 cases/816 controls) | Premenopausal women: 1.3,( 0.9-2)  Postmenopausal women: 0.7,( 0.4-1.2) | 1,4,6,24,2,3 |  | 8 |  |
| Catherine Schairer(71)  2020 | | a nested case-control study | Amrica | Females=247  CONTROL=2470  AGE= 25-95 | (41cases/252 controls)  Postmenopausal women: (192cases/192 controls) | OR=2.3,( 1.53-3.45)  Postmenopausal women: 2.9,( 1.4-6) |  | Inflammatory breast cancer | 9 |  |
| Xin Wang(72)  2016 | | China | case-control | Total CASE=1400  Age=47.64±8.70  CONTROL=1400  Age=47.01±8.72 | Premenopausal=  Case=465  Control=402  Postmenopausal=  Case=894  Control=946 | Premenopausal women: 1.09,( 0.76-1.57)  Postmenopausal women: 1.82,( 1.33-2.5) | 4,6,8,25 |  | 9 |  |
| Wambui G. Gathirua-Mwangi(73)  2015 | | U.A | Case-control | 2,895 women:  172 with breast  Cancer  Age mean=67.6  2,723 without breast cancer  Age mean=63.5 | (6 cases/150 controls) | OR=1.12,( 0.76-1.65) | 1,14,11,23,4,5,16 |  | 7 |  |
| Karla Kerlikowske(74)  2008 | | USA | Case-control? | 287 115 postmenopausal  aged 40 years and older | (780cases/1617controls) | postmenopausal OR=1.2,( 1.1-1.31) | 1,23 |  |  |  |
| Naser Elkum(75)  2014 | | Saudi Arabia | case-control | Arab women:  534 cases:  Pre-menopause =266  Post-menopause =267  638 controls | All:  (248cases/198 controls) | Premenopausal women: 2.73 (1.79 – 4.18)  Postmenopausal women: 2.22 (1.32 – 3.72)  All: 2.4,( 1.81-3.18) | 1,14,31,34,4,8,11 |  | 8 |  |
| Laurence N. Kolonel(76)  2009 | | USA | Case-control | 138 Japanese postmenopausal cases, 134 Caucasian postmenopausal cases, 154 Japanese  neighborhood controls, and 142 Caucasian neighborhood control  age=45-74 | (138case/154controls) | Postmenopausal women: 1.15,(0.69-1.92) | 1,6,3,2, |  | 9 |  |
| Carlos Nunez(76)  2017 | | Australia | Case-control | All cancer=8823 participants (6831 self-reported cancer cases of any type and 1992 self-reported cancer free controls)  Breast cancer in women:  Control=1031  Maen age=58.3 | postmenopausal women : ( 364cases/210  Controls) | OR=1.04(0.83–1.30) | 1,26 |  |  |  |
| TienYu Owen Yang(77)(2022) | | UK | Cohort | 342079 postmenopausal UK women (average age at completion 61 years [SD 5]) | 15,506 breast cancer | OR=1.20 (1.18-1.22) | 11,12,1,16,25,26,4,10,24,7,5 |  | 8 |  |
| Mokhtar Hamdi-Cherif(78) (2020) | | Algeria | Case -control | 1227 (age >40) | (612 cases/615 controls) | OR= 1.04 (0.74-1.45) | 1,11 |  | 8 |  |
| Andrew G. Renehan(79) (2020) | | UK | Cohort | 131,373 women aged 46–73 years | Post-menopausal:  (703expose/31449cnon expose)  Pre-/peri-menopausal: (413expose/14451non expose | Post-menopausal: HR= 0.866 (0.794, 0.945)  Pre-/peri menopausal: HR= 0.919 (0.800, 1.056) | 1,14,4,23,31,25,26,9,10 |  | 6 |  |
| Mathilde His (80) (2020) | | Latin America | Case- control | 812 women aged 20–45 | premenopausal women :(cases 406/406 controls) | OR= 0.36 (0.22–0.59) | 11,2,3,26,10,9,8,4,16,25,36 |  | 7 |  |
| Marie Klintman(81)  (2022) | | Sweden | Cohort | 35,412 ( Median age at baseline was 62 years) | (12029 expose/23383non expose) | HR=1.19 (0.94–1.50) | 4,7,9,16,25 |  | 7 |  |
| Jae Won Park(82) (2021) | | Korea | cohort | 6,467,388 (women≥40years) | Post-menopausal:  ( 2138expose/ 106,421non expose)  Premenopausal: (536expose/ 51,527non expose) | Pre-menopause:  HR=0.90 (0.82,0.98)  Post -menopause:  HR= 1.54 (1.47,1.62) | 1,16,24,25,26,8,7,4,5 |  | 8 |  |
| Farzad Maleki(83)(2020) | | Iran | case–control | 1925 females (at least 18 years of age) | (958 cases /967 controls) | Premenopausal:  OR=1.07 (0.73, 1.57)  Postmenopausal:  OR=1.90 (1.14, 3.14) | 1,4,11,24,8,7,26,6 |  | 7 |  |
| Islam, D. (84) (2022) | | Bangladesh | case-control | 111 (adult females between 25 and 70 years of age) | (52 cases/ 59 controls) | OR= 3.91(1.00-15.31) |  |  | 6 |  |
| P. M. Y. Lee (2020) (85) | | Hong Kong  China | case-control | 20– 84 years old  Females | (923 cases/1013Controls) | OR=1.25,( 1.14, 1.37) | 1,2,4,6,24,8,34 |  | 6 |  |
| E. R. Miller (2018) (86) | | Australia | case control | 45 – 64 years old  females | Cases (n = 80) controls (n = 235) | OR =3.5, ( 1.3–9.4). | - |  |  |  |
| Y. Gao (2016) (87) | | China | cross sectinal | 397 thousand urban women aged 35–69 years old  813 thousand rural women aged 35–59 | (22 cases/ 22 625 controls) | OR=2.05,( 1.3 -3.23) |  |  |  |  |
| N. Elkum (2014) (88) | | Saudi Arabia | case-control | Females  aged 18 years or older | Cases= 534  Controls= 638 | OR= 2.29 (1.68 – 3.13) | 2,7,11,8,16 |  | 8 |  |
| F. Z. Laamiri (2014) (89) | | Morocco | case-control | Females  22-75 years old | Cases= 400  Controls= 400 | OR =1.30 (1.25-1.37) | 1 |  | 8 |  |
| M. Minatoya (2014) (90) | | Japan | case-control | Females | Cases= 66  Controls= 66 | Premenopausal:  OR=1.17 (0.23-6.10)  Postmenopausal:  OR= 1.39,( 0.5-3.86) | 4,16,25,7,24 |  | 7 |  |
| S. Sangrajrang (2013) (91) | | Thailand | Case-control | Females  mean age of controls (43.7±11.6 years)  , cases  (47.0±10.4 years) | Cases= 1130  Controls=1142 | All= 1.33  Premenopausal=  1.22,( 0.73-2.03)  Postmenopausal=  1.32,( 0.77-2.26) | 1,2,11,25,16,8,29 |  | 8 |  |
| M. Sarkissyan (2011) (92) | | U.S | cross-sectional | Females | Cases=237  Controls= 234 | OR=2.02,( 1.39- 2.92) |  |  | 6 |  |
| Fokhrul M. Hossain (93)(2022) | | Louisiana | Case-control | Females | Luminal A: (1884 cases/5436 controls)  Luminal B: (232cases/ 1,095 controls)  TNBC: (364 cases/ 1,686 controls)  HER2: (115case/562 controls) | OR= 1.06(0.92, 1.23) | 1,36,23 | Luminal A,  Luminal B,  TNBC,  HER2: | 7 |  |
| Takaaki Konish(94)  (2022) | | Japan | retrospective cohort | 1,032,639 females aged < 45 years | Premenopausal (75 cases/618 controls) | Premenopausal=  0.78 (0.62–  0.98) | 1,16,25,26 |  | 7 |  |
| Kedma Anne Lima Gomes (95) (2022) | | Brazil | case-control | women in Brazilian populations | (313 cases/321 controls) | Total :1.69 (95% CI: 1.08–2.63)  Postmenopausal= 2.02 (95% CI: 1.22–3.37) | 2,25,7 | uminal A: Luminal B: HER2/neu: triple-negative breast cancer (TNBC): | - |  |
| Aivara Urbute(96) 2022 | | Danish | cohort | 461,646 women |  | Premenopausal:  HR = 0.77, ( 0.68–0.87) | 24,7,2,1,11,16 |  |  |  |
| Wambui G Gathirua-Mwangi(97) (2022) | | African Americans | cohort | Black Women’s Health Study | 1,384 cases | Postmenopausal= HR=1.42 (1.10, 1.84) | - | 787 estrogen-receptor positive (ER+) cases and 310 ER-negative cases |  |  |
| Tara M. Friebel‑Klingner (98)(2021) | | Pennsylvania | cohort | Black women who underwent screening mammography between the ages of 40–84 years | ER/PR+HER2−N=218  Triple negative N=61 | OR=1.54,( 1.04-2.28) | 1,4,6,2 |  | 8 |  |
| Mariana Brandão (99)(2021) | | Africa | cohort | 138cases/638 controls | Premenopausal=(25 cases/89 controls)  Postmenopausal=(26cases/48 controls) | Premenopausal=  0.69 (0.37–1.28)  Postmenopausal=  1.73 (0.84–3.60) | 11,12,9,,1 |  | 8 |  |
| Jae Won Park (100)(2021) | | Korean | cohort | 6,467,388 women≥40 years | Premenopausal (48,033 cases/212,943controls)  Postmenopausal( 212,943cases/ 106,434contols) | Premenopausal=  0.90, (0.82–0.98)  Postmenopausal= 1.54,(1.47–1.62) | 1,16,25,24,26,8,7,4 |  | 9 |  |
| Farzad Maleki (101) (2020) | | Iran | case–control | 958 BC cases with 967 controls | Premenopausal: (211cases/196controls)  Postmenopausal: (181cases/152controls) | Postmenopausal:  OR = 1.9; ( 1.14, 3.14)  Premenopausal: 1.07 (0.73, 1.57) | 1,4,11,24,8,7,26,6 |  | 9 |  |
| Hwayoung Noh (102)(2020) | | Icelandic | Cohort | 58,454 women cancer aged 50 years or older | (cases n = 2,718) | Postmenopausal:  OR = 0.99 (0.82, 1.18) | 1,12,11,4,8,7,24 |  | 8 |  |

1-age;2- family history of breast cancer;3- history of benign breast disease,4-age at menarche,5- age at menopause;6-age at first birth, 7-Oc use;8-breastfeeding (yes/no), 9- number of births ;10-age at first full trem, 11-education; 12-height, 13- region,14- having a partner (yes or no), 15- waist circumference,16-smoking status (never, former, current), 17-tumor size, 18-clinical stage,19-nuclear grade,20- grade,21-hormone receptors, 22- HER2 expression, 23- ethnicity,24- parity,25- alcohol drinking, 26-physical activity, 27-comorbidities, 28-use of NSAIDs, 29- number of miscarriage, 30-total life satisfaction,31- hormon traphy,32- caloric intake per day, 33- weight at 18 years, 34-menopasaul statuse,35- lacration,36-diabetes

1. Nattenmüller CJ, Kriegsmann M, Sookthai D, Fortner RT, Steffen A, Walter B, et al. Obesity as risk factor for subtypes of breast cancer: results from a prospective cohort study. BMC cancer. 2018;18(1):1-8.

2. Arce-Salinas C, Aguilar-Ponce J, Villarreal-Garza C, Lara-Medina F, Olvera-Caraza D, Alvarado Miranda A, et al. Overweight and obesity as poor prognostic factors in locally advanced breast cancer patients. Breast cancer research and treatment. 2014;146(1):183-8.

3. Bandera EV, Chandran U, Zirpoli G, Gong Z, McCann SE, Hong C-C, et al. Body fatness and breast cancer risk in women of African ancestry. BMC cancer. 2013;13(1):1-13.

4. Chen M, Wu WY, Yen AM, Fann JC, Chen SL, Chiu SY, et al. Body mass index and breast cancer: analysis of a nation-wide population-based prospective cohort study on 1 393 985 Taiwanese women. International Journal of Obesity. 2016;40(3):524-30.

5. Fang Q, Tong Y-W, Wang G, Zhang N, Chen W-G, Li Y-F, et al. Neutrophil-to-lymphocyte ratio, obesity, and breast cancer risk in Chinese population. Medicine. 2018;97(30).

6. Haakinson DJ, Leeds SG, Dueck AC, Gray RJ, Wasif N, Stucky C-CH, et al. The impact of obesity on breast cancer: a retrospective review. Annals of surgical oncology. 2012;19(9):3012-8.

7. Keinan-Boker L, Levine H, Derazne E, Molina-Hazan V, Kark JD. Measured adolescent body mass index and adult breast cancer in a cohort of 951,480 women. Breast cancer research and treatment. 2016;158(1):157-67.

8. Kops NL, Bessel M, Caleffi M, Ribeiro RA, Wendland EM. Body Weight and Breast Cancer: Nested Case–Control Study in Southern Brazil. Clinical Breast Cancer. 2018;18(5):e797-e803.

9. Maskarinec G, Jacobs S, Park S-Y, Haiman CA, Setiawan VW, Wilkens LR, et al. Type II Diabetes, Obesity, and Breast Cancer Risk: The Multiethnic CohortDiabetes, Obesity, and Breast Cancer. Cancer Epidemiology, Biomarkers & Prevention. 2017;26(6):854-61.

10. Nagrani R, Mhatre S, Rajaraman P, Soerjomataram I, Boffetta P, Gupta S, et al. Central obesity increases risk of breast cancer irrespective of menopausal and hormonal receptor status in women of South Asian Ethnicity. European Journal of Cancer. 2016;66:153-61.

11. Naushad SM, Hussain T, Al-Attas OS, Prayaga A, Digumarti RR, Gottumukkala SR, et al. Molecular insights into the association of obesity with breast cancer risk: relevance to xenobiotic metabolism and CpG island methylation of tumor suppressor genes. Molecular and cellular biochemistry. 2014;392(1):273-80.

12. Phipps AI, Buist DS, Malone KE, Barlow WE, Porter PL, Kerlikowske K, et al. Breast density, body mass index, and risk of tumor marker-defined subtypes of breast cancer. Annals of epidemiology. 2012;22(5):340-8.

13. Smith A, Mullooly M, Murphy L, Barron TI, Bennett K. Associations between obesity, smoking and lymph node status at breast cancer diagnosis in the Prostate, Lung, Colorectal and Ovarian (PLCO) Cancer Screening Trial. Plos one. 2018;13(8):e0202291.

14. Suzuki S, Kojima M, Tokudome S, Mori M, Sakauchi F, Wakai K, et al. Obesity/weight gain and breast cancer risk: findings from the Japan collaborative cohort study for the evaluation of cancer risk. Journal of epidemiology. 2013:JE20120102.

15. Tamaki K, Tamaki N, Terukina S, Kamada Y, Uehara K, Arakaki M, et al. The correlation between body mass index and breast cancer risk or estrogen receptor status in Okinawan women. The Tohoku Journal of Experimental Medicine. 2014;234(3):169-74.

16. Wada K, Nagata C, Tamakoshi A, Matsuo K, Oze I, Wakai K, et al. Body mass index and breast cancer risk in Japan: a pooled analysis of eight population-based cohort studies. Annals of oncology. 2014;25(2):519-24.

17. Wang X-L, Jia C-X, Liu L-Y, Zhang Q, Li Y-Y, Li L. Obesity, diabetes mellitus, and the risk of female breast cancer in Eastern China. World Journal of Surgical Oncology. 2013;11(1):1-7.

18. White KK, Park SY, Kolonel LN, Henderson BE, Wilkens LR. Body size and breast cancer risk: the Multiethnic Cohort. International journal of cancer. 2012;131(5):E705-E16.

19. Baset Z, Abdul-Ghafar J, Parpio YN, Haidary AM. Risk factors of breast cancer among patients in a tertiary care hospitals in Afghanistan: a case control study. BMC cancer. 2021;21:1-9.

20. Khan A, Rafique K, Farooq U, Khan K. Obesity, fat topography and risk of carcinoma breast. Journal of Ayub Medical College Abbottabad. 2017;29(3):419-21.

21. Li H-T, Han X-H, Liu Y-X, Leng K-M, Dong G-M. Relationship between body mass index and incidence of breast cancer. International journal of clinical and experimental medicine. 2015;8(7):11549.

22. Miyagawa Y, Miyake T, Yanai A, Murase K, Imamura M, Ichii S, et al. Association of body mass index with risk of luminal A but not luminal B estrogen receptor-positive and HER2-negative breast cancer for postmenopausal Japanese women. Breast cancer. 2015;22:399-405.

23. Cao S, Zhou J, Zhu Z, Wei F, Li W, Lu S, et al. Adult weight change and the risk of pre-and postmenopausal breast cancer in the Chinese Wuxi Exposure and Breast Cancer Study. Breast cancer research and treatment. 2019;173:647-55.

24. Shi J, Zhang M, Li L, Holman C, Chen J, Teng Y, et al. Body mass index and its change in adulthood and breast cancer risk in China. Asian Pac J Cancer Prev. 2010;11(5):1213-8.

25. Tian Y-F, Chu C-H, Wu M-H, Chang C-L, Yang T, Chou Y-C, et al. Anthropometric measures, plasma adiponectin, and breast cancer risk. Endocrine-related cancer. 2007;14(3):669-77.

26. Verla-Tebit E, Chang-Claude J. Anthropometric factors and the risk of premenopausal breast cancer in Germany. European journal of cancer prevention. 2005:419-26.

27. John EM, Sangaramoorthy M, Hines LM, Stern MC, Baumgartner KB, Giuliano AR, et al. Body Size Throughout Adult Life Influences Postmenopausal Breast Cancer Risk among Hispanic Women: The Breast Cancer Health Disparities StudyBody Size and Postmenopausal Breast Cancer. Cancer Epidemiology, Biomarkers & Prevention. 2015;24(1):128-37.

28. Ogundiran TO, Huo D, Adenipekun A, Campbell O, Oyesegun R, Akang E, et al. Body fat distribution and breast cancer risk: findings from the Nigerian breast cancer study. Cancer causes & control. 2012;23:565-74.

29. Chow LW, Lui KL, Chan JCY, Chan TC, Ho PK, Lee WY, et al. Association between body mass index and risk of formation of breast cancer in Chinese women. Asian Journal of Surgery. 2005;28(3):179-84.

30. Morimoto LM, White E, Chen Z, Chlebowski RT, Hays J, Kuller L, et al. Obesity, body size, and risk of postmenopausal breast cancer: the Women's Health Initiative (United States). Cancer Causes & Control. 2002;13:741-51.

31. Shu XO, Jin F, Dai Q, Shi JR, Potter JD, Brinton LA, et al. Association of body size and fat distribution with risk of breast cancer among Chinese women. International journal of cancer. 2001;94(3):449-55.

32. Yoo K-Y, Tajima K, Park S-K, Kang D, Kim S-U, Hirose K, et al. Postmenopausal obesity as a breast cancer risk factor according to estrogen and progesterone receptor status (Japan). Cancer letters. 2001;167(1):57-63.

33. Li CI, Stanford JL, Daling JR. Anthropometric variables in relation to risk of breast cancer in middle-aged women. International journal of epidemiology. 2000;29(2):208-13.

34. Hall IJ, Newman B, Millikan RC, Moorman PG. Body size and breast cancer risk in black women and white women: the Carolina Breast Cancer Study. American journal of epidemiology. 2000;151(8):754-64.

35. Peacock SL, White E, Daling JR, Voigt LF, Malone KE. Relation between obesity and breast cancer in young women. American journal of epidemiology. 1999;149(4):339-46.

36. Kaaks R, Van Noord PA, Den Tonkelaar I, Peeters PH, Riboli E, Grobbee DE. Breast‐cancer incidence in relation to height, weight and body‐fat distribution in the Dutch “DOM” cohort. International journal of cancer. 1998;76(5):647-51.

37. Ng EH, Gao F, Ji CY, Ho GH, Soo KC. Risk factors for breast carcinoma in Singaporean Chinese women: the role of central obesity. Cancer: Interdisciplinary International Journal of the American Cancer Society. 1997;80(4):725-31.

38. Franceschi S, Favero A, La Vecchia C, Barón AE, Negri E, Dal Maso L, et al. Body size indices and breast cancer risk before and after menopause. International journal of cancer. 1996;67(2):181-6.

39. Swanson CA, Coates RJ, Schoenberg JB, Malone KE, Gammon MD, Stanford JL, et al. Body size and breast cancer risk among women under age 45 years. American journal of epidemiology. 1996;143(7):698-706.

40. Ursin G, Paganini-Hill A, Siemiatycki J, Thompson WD, Haile RW. Early adult body weight, body mass index, and premenopausal bilateral breast cancer: data from a case-control study. Breast cancer research and treatment. 1995;33:75-82.

41. Folsom AR, Kaye SA, Prineas RJ, Potter JD, Gapstur SM, Wallace RB. Increased incidence of carcinoma of the breast associated with abdominal adiposity in postmenopausal women. American journal of epidemiology. 1990;131(5):794-803.

42. Khalis M, Dossus L, Rinaldi S, Biessy C, Moskal A, Charaka H, et al. Body size, silhouette trajectory and the risk of breast cancer in a Moroccan case–control study. Breast Cancer. 2020;27:748-58.

43. Taleban R, Sirous R, Sirous M, Razavi S, Taghvaei R, Sirous S, et al. The relationship between anthropometric indices and breast cancer in central Iran. Nutrition and cancer. 2019;71(8):1276-82.

44. Gravena AAF, Lopes TCR, de Oliveira Demitto M, Borghesan DHP, Dell’Agnolo CM, Brischiliari SCR, et al. The obesity and the risk of breast cancer among pre and postmenopausal women. Asian Pacific journal of cancer prevention: APJCP. 2018;19(9):2429.

45. Chaveepojnkamjorn W, Thotong R, Sativipawee P, Pitikultang S. Body mass index and breast cancer risk among Thai premenopausal women: A case-control study. Asian Pacific Journal of Cancer Prevention: APJCP. 2017;18(11):3097.

46. Pacholczak R, Klimek-Piotrowska W, Kuszmiersz P. Associations of anthropometric measures on breast cancer risk in pre-and postmenopausal women—a case-control study. Journal of Physiological Anthropology. 2016;35(1):1-10.

47. Bandera EV, Chandran U, Hong C-C, Troester MA, Bethea TN, Adams-Campbell LL, et al. Obesity, body fat distribution, and risk of breast cancer subtypes in African American women participating in the AMBER Consortium. Breast cancer research and treatment. 2015;150:655-66.

48. Ma H, Ursin G, Xu X, Lee E, Togawa K, Malone KE, et al. Body mass index at age 18 years and recent body mass index in relation to risk of breast cancer overall and ER/PR/HER2-defined subtypes in white women and African-American women: a pooled analysis. Breast Cancer Research. 2018;20(1):1-14.

49. His M, Biessy C, Torres-Mejía G, Ángeles-Llerenas A, Alvarado-Cabrero I, Sánchez GI, et al. Anthropometry, body shape in early-life and risk of premenopausal breast cancer among Latin American women: results from the PRECAMA study. Scientific Reports. 2020;10(1):1-12.

50. Charkhchi P, Schabath MB, Carlos RC. Breast, cervical, and colorectal cancer screening adherence: effect of low body mass index in women. Journal of Women's Health. 2020;29(7):996-1006.

51. Jeong SH, An Y, Ahn C, Park B, Lee MH, Noh D-Y, et al. Body mass index and risk of breast cancer molecular subtypes in Korean women: a case–control study. Breast Cancer Research and Treatment. 2020;179:459-70.

52. Singh P, Kapil U, Shukla N, Deo S, Dwivedi S. Association of overweight and obesity with breast cancer in India. Indian journal of community medicine: official publication of Indian Association of Preventive & Social Medicine. 2011;36(4):259.

53. Montazeri A, Sadighi J, Farzadi F, Maftoon F, Vahdaninia M, Ansari M, et al. Weight, height, body mass index and risk of breast cancer in postmenopausal women: a case-control study. BMC cancer. 2008;8:1-7.

54. Phipps AI, Malone KE, Porter PL, Daling JR, Li CI. Body size and risk of luminal, HER2-overexpressing, and triple-negative breast cancer in postmenopausal women. Cancer Epidemiology Biomarkers & Prevention. 2008;17(8):2078-86.

55. Yumuk PF, Dane F, Yumuk V, Yazici D, Ege B, Bekiroglu N, et al. Impact of body mass index on cancer development. Journal of BU ON: official journal of the Balkan Union of Oncology. 2008;13(1):55-9.

56. Slattery ML, Sweeney C, Edwards S, Herrick J, Baumgartner K, Wolff R, et al. Body size, weight change, fat distribution and breast cancer risk in Hispanic and non-Hispanic white women. Breast cancer research and treatment. 2007;102:85-101.

57. Mellemkjær L, Bigaard J, Tjønneland A, Christensen J, Thomsen B, Johansen C, et al. Body composition and breast cancer in postmenopausal women: a Danish prospective cohort study. Obesity. 2006;14(10):1854-62.

58. Park JK, Park HA, Park JJ, Cho YG. Obesity and screening compliance for breast and cervical cancer in Korean women. Asian Pacific Journal of Cancer Prevention. 2012;13(7):3271-4.

59. Sarkissyan M, Wu Y, Vadgama JV. Obesity is associated with breast cancer in African‐American women but not Hispanic women in South Los Angeles. Cancer. 2011;117(16):3814-23.

60. Nemesure B, Wu S-Y, Hennis A, Leske MC, Group BNCS. Body size and breast cancer in a black population—the Barbados National Cancer Study. Cancer Causes & Control. 2009;20:387-94.

61. Máchová L, Čížek L, Horakova D, Koutná J, Lorenc J, Janoutova G, et al. Association between obesity and cancer incidence in the population of the District Sumperk, Czech Republic. Oncology Research and Treatment. 2007;30(11):538-42.

62. Amadou A, Mejia GT, Fagherazzi G, Ortega C, Angeles-Llerenas A, Chajes V, et al. Anthropometry, silhouette trajectory, and risk of breast cancer in Mexican women. American Journal of Preventive Medicine. 2014;46(3):S52-S64.

63. Gaudet MM, Carter BD, Patel AV, Teras LR, Jacobs EJ, Gapstur SM. Waist circumference, body mass index, and postmenopausal breast cancer incidence in the Cancer Prevention Study-II Nutrition Cohort. Cancer Causes & Control. 2014;25:737-45.

64. Canchola AJ, Anton-Culver H, Bernstein L, Clarke CA, Henderson K, Ma H, et al. Body size and the risk of postmenopausal breast cancer subtypes in the California Teachers Study cohort. Cancer Causes & Control. 2012;23:473-85.

65. Kuriyama S, Tsubono Y, Hozawa A, Shimazu T, Suzuki Y, Koizumi Y, et al. Obesity and risk of cancer in Japan. International journal of cancer. 2005;113(1):148-57.

66. Lahmann PH, Hoffmann K, Allen N, Van Gils CH, Khaw KT, Tehard B, et al. Body size and breast cancer risk: findings from the European Prospective Investigation into Cancer And Nutrition (EPIC). International journal of cancer. 2004;111(5):762-71.

67. Pan SY, Johnson KC, Ugnat A-M, Wen SW, Mao Y. Association of obesity and cancer risk in Canada. American journal of epidemiology. 2004;159(3):259-68.

68. Jonsson F, Wolk A, Pedersen NL, Lichtenstein P, Terry P, Ahlbom A, et al. Obesity and hormone‐dependent tumors: Cohort and co‐twin control studies based on the Swedish Twin Registry. International journal of cancer. 2003;106(4):594-9.

69. Wenten M, Gilliland FD, Baumgartner K, Samet JM. Associations of weight, weight change, and body mass with breast cancer risk in Hispanic and non-Hispanic white women. Annals of epidemiology. 2002;12(6):435-44.

70. Chu SY, Lee NC, Wingo PA, Senie RT, Greenberg RS, Peterson HB. The relationship between body mass and breast cancer among women enrolled in the Cancer and Steroid Hormone Study. Journal of clinical epidemiology. 1991;44(11):1197-206.

71. Schairer C, Laurent CA, Moy LM, Gierach GL, Caporaso NE, Pfeiffer RM, et al. Obesity and related conditions and risk of inflammatory breast cancer: A nested case–control study. Breast cancer research and treatment. 2020;183:467-78.

72. Wang X, Li L, Gao J, Liu J, Guo M, Liu L, et al. The association between body size and breast cancer in Han women in Northern and Eastern China. The Oncologist. 2016;21(11):1362-8.

73. Gathirua-Mwangi WG, Zollinger TW, Murage MJ, Pradhan KR, Champion VL. Adult BMI change and risk of breast cancer: National Health and Nutrition Examination Survey (NHANES) 2005–2010. Breast cancer. 2015;22:648-56.

74. Kerlikowske K, Walker R, Miglioretti DL, Desai A, Ballard-Barbash R, Buist DS, et al. Obesity, mammography use and accuracy, and advanced breast cancer risk. JNCI: Journal of the National Cancer Institute. 2008;100(23):1724-33.

75. Elkum N, Al-Tweigeri T, Ajarim D, Al-Zahrani A, Amer SMB, Aboussekhra A. Obesity is a significant risk factor for breast cancer in Arab women. BMC cancer. 2014;14(1):1-10.

76. Kolonel LN, Nomura AM, Lee J, Hirohata T. Anthropometric indicators of breast cancer risk in postmenopausal women in Hawaii. 1986.

77. Yang TO, Cairns BJ, Pirie K, Green J, Beral V, Floud S, et al. Body size in early life and the risk of postmenopausal breast cancer. BMC cancer. 2022;22(1):232.

78. Hamdi-Cherif M, Serraino D, Bouaoud S, Dib A, Boudaoud K, Atoui S, et al. Sociodemographic and reproductive risk factors for breast cancer: A case-control study in the Setif Province, Northern Algeria. Asian Pacific journal of cancer prevention: APJCP. 2020;21(2):457.

79. Renehan AG, Pegington M, Harvie MN, Sperrin M, Astley SM, Brentnall AR, et al. Young adulthood body mass index, adult weight gain and breast cancer risk: the PROCAS Study (United Kingdom). British journal of cancer. 2020;122(10):1552-61.

80. His M, Biessy C, Torres-Mejía G, Ángeles-Llerenas A, Alvarado-Cabrero I, Sánchez GI, et al. Anthropometry, body shape in early-life and risk of premenopausal breast cancer among Latin American women: results from the PRECAMA study. Scientific reports. 2020;10(1):2294.

81. Klintman M, Rosendahl AH, Randeris B, Eriksson M, Czene K, Hall P, et al. Postmenopausal overweight and breast cancer risk; results from the KARMA cohort. Breast cancer research and treatment. 2022;196(1):185-96.

82. Park JW, Han K, Shin DW, Yeo Y, Chang JW, Yoo JE, et al. Obesity and breast cancer risk for pre- and postmenopausal women among over 6 million Korean women. Breast cancer research and treatment. 2021;185(2):495-506.

83. Maleki F, Fotouhi A, Ghiasvand R, Harirchi I, Talebi G, Rostami S, et al. Association of physical activity, body mass index and reproductive history with breast cancer by menopausal status in Iranian women. Cancer epidemiology. 2020;67:101738.

84. Islam D, Islam MS, Jesmin. Association of hypertension, hyperlipidemia, obesity, and demographic risk factors with breast cancer in Bangladeshi women. Medicine. 2022;101(46):e31698.

85. Lee PMY, Kwok CH, Chan WC, Wu C, Tsang KH, Law SH, et al. Heterogeneous Associations Between Obesity and Reproductive-Related Factors and Specific Breast Cancer Subtypes Among Hong Kong Chinese Women. Horm Cancer. 2020;11(3-4):191-9.

86. Miller ER, Wilson C, Chapman J, Flight I, Nguyen AM, Fletcher C, et al. Connecting the dots between breast cancer, obesity and alcohol consumption in middle-aged women: ecological and case control studies. BMC Public Health. 2018;18(1):460.

87. Gao Y, Huang Y, Song F, Dai H, Wang P, Li H, et al. Urban-rural disparity of overweight/obesity distribution and its potential trend with breast cancer among Chinese women. Oncotarget. 2016;7(35):56608-18.

88. Elkum N, Al-Tweigeri T, Ajarim D, Al-Zahrani A, Amer SM, Aboussekhra A. Obesity is a significant risk factor for breast cancer in Arab women. BMC Cancer. 2014;14:788.

89. Laamiri FZ, Bouayad A, Otmani A, Ahid S, Mrabet M, Barkat A. Dietery factor obesity microenvironnement and breast cancer. Gland Surg. 2014;3(3):165-73.

90. Minatoya M, Kutomi G, Shima H, Asakura S, Otokozawa S, Ohnishi H, et al. Relation of serum adiponectin levels and obesity with breast cancer: a Japanese case-control study. Asian Pac J Cancer Prev. 2014;15(19):8325-30.

91. Sangrajrang S, Chaiwerawattana A, Ploysawang P, Nooklang K, Jamsri P, Somharnwong S. Obesity, diet and physical inactivity and risk of breast cancer in Thai women. Asian Pac J Cancer Prev. 2013;14(11):7023-7.

92. Sarkissyan M, Wu Y, Vadgama JV. Obesity is associated with breast cancer in African-American women but not Hispanic women in South Los Angeles. Cancer. 2011;117(16):3814-23.

93. Hossain FM, Danos DM, Fu Q, Wang X, Scribner RA, Chu ST, et al. Association of obesity and diabetes with the incidence of breast cancer in louisiana. American Journal of Preventive Medicine. 2022;63(1):S83-S92.

94. Konishi T, Fujiogi M, Michihata N, Matsui H, Tanabe M, Seto Y, et al. Association between body mass index and incidence of breast cancer in premenopausal women: a Japanese nationwide database study. Breast Cancer Research and Treatment. 2022;194(2):315-25.

95. Gomes KAL, de Araújo Jerônimo AF, Guimarães CMC, de Oliveira Ramos R, dos Santos Andrade LS, Weller M. Risk factors for breast cancer and their association with molecular subtypes in a population of Northeast Brazil. Cancer Epidemiology. 2022;78:102166.

96. Urbute A, Frederiksen K, Kjaer SK. Early adulthood overweight and obesity and risk of premenopausal ovarian cancer, and premenopausal breast cancer including receptor status: prospective cohort study of nearly 500,000 Danish women. Annals of Epidemiology. 2022;70:61-7.

97. Gathirua-Mwangi WG, Palmer JR, Champion V, Castro-Webb N, Stokes AC, Adams-Campbell L, et al. Maximum and time-dependent body mass index and breast cancer incidence among postmenopausal women in the black women’s health study. American journal of epidemiology. 2022;191(4):646-54.

98. Friebel-Klingner TM, Ehsan S, Conant EF, Kontos D, Domchek SM, McCarthy AM. Risk factors for breast cancer subtypes among Black women undergoing screening mammography. Breast Cancer Research and Treatment. 2021;189(3):827-35.

99. Brandão M, Guisseve A, Damasceno A, Bata G, Silva-Matos C, Alberto M, et al. Risk factors for breast cancer, overall and by tumor subtype, among women from Mozambique, Sub-Saharan Africa. Cancer Epidemiology, Biomarkers & Prevention. 2021;30(6):1250-9.

100. Park JW, Han K, Shin DW, Yeo Y, Chang JW, Yoo JE, et al. Obesity and breast cancer risk for pre-and postmenopausal women among over 6 million Korean women. Breast cancer research and treatment. 2021;185:495-506.

101. Maleki F, Fotouhi A, Ghiasvand R, Harirchi I, Talebi G, Rostami S, et al. Association of physical activity, body mass index and reproductive history with breast cancer by menopausal status in Iranian women. Cancer Epidemiology. 2020;67:101738.

102. Noh H, Charvat H, Freisling H, Ólafsdóttir GH, Ólafsdóttir EJ, Tryggvadóttir L, et al. Cumulative exposure to premenopausal obesity and risk of postmenopausal cancer: a population‐based study in Icelandic women. International Journal of Cancer. 2020;147(3):793-802.
